# Supplementary material for: Content-rich biological network constructed by mining PubMed abstracts
Source: BMC Bioinformatics. 2004 Oct 8;5:147. doi: 10.1186/1471-2105-5-147 (PMC528731; doi:10.1186/1471-2105-5-147)
Supplement: Additional File 2 — The original results of the above study (non-essential files are deleted to keep the file size under the limit set by BMC bioinformatics). [file 1471-2105-5-147-S2.bz2 › chilibotAdditionalFile2/dip40/366ID9045692E6040/html/FGF1_FGFR3.html]

 


 **FGF1** and **FGFR3** 
  
Found 170 abstracts in PubMed, retrieved 40.  
 

 What does Google say? 
 PDF only 
| .edu only 

---

**Interactive relationship** (e.g. stimulation, inhibition, etc)

**Neutral relationship**- These findings suggest a proteolytic regulatory mechanism for  **FGFR3** , whereby  **Fgfr3**  fragments could control availability of FGF  [ **FGF1** ]  for the intact receptor, and by which proteolysis could inactivate the receptor.  Ref: 11772395 Biochem J, 2002
- 2 to determine the distribution, in the mature mammalian cochlea, of FGF 1  [ **FGF1** ]  and the receptor,  **FGFR3** , to which it binds with high affinity.  Ref: 12512896 J Otolaryngol, 2002
- Sf9 cells transfected with the corresponding cDNA express the soluble form of  **FGFR3**  AT III into the condition medium and its secreted form was able to bind both FGF 1  [ **FGF1** ]  and FGF  [ **FGF1** ]  2 leading to loss of ligand binding specificity.  Ref: 11906172 Biochem Biophys Res Commun, 2002
- Forced expression of  **FGFR3**  in C3H10T1 2 is sufficient for chondrogenic differentiation, indicating an important role for FGF  [ **FGF1** ]  signaling during the manifestation of the chondrogenic lineage in this cell line.  Ref: 11865033 J Cell Sci, 2002
- The findings show that the surgically created lesions contain only a small amount of FGF  [ **FGF1** ]  receptor 3  **FGFR3**  expressed on mesenchymal stem cells located in the perichondrium, as compared to the cell population carrying  **FGFR3**  in the contralateral limb.  Ref: 12645631 Exp Mol Pathol, 2003
- These observations coupled with our observation that multiple avian FGF  [ **FGF1** ]  receptors Cek1, Cek2  [ **FGFR3** ] , Cek3, and FREK are expressed in proliferative chondrocytes highlights the complexity of FGF  [ **FGF1** ]  signaling pathways in postnatal endochondral bone growth.  Ref: 11787065 J Cell Biochem, 2002
- Alternative splicing of the  **Fgfr3**  transcript gives rise to two isoforms, IIIc and IIIb, which vary in their specificity for FGF  [ **FGF1** ]  ligands.  Ref: 11772395 Biochem J, 2002
- Importantly, we also find a number of gene expression changes indicating that FGF  [ **FGF1** ]  promotes many aspects of hypertrophic differentiation, a notion supported by in situ analysis of developing growth plates from mice expressing an activated form of  **FGFR3** .  Ref: 12821644 J Cell Biol< MedlineTA>J Cell Biol, 2002
- Wild type  **FGFR3**  became dimerized and phosphorylated only on FGF  [ **FGF1** ]  stimulation.  Ref: 12009017 J Bone Miner Res, 2002

**Non-interactive relationship** (e.g. studied together, co-existance, homology, etc.)

- Immunoreactivity for receptors R for IGF 1 and FGF  [ **FGF1** ]  2 IGF 1R, FGFR1, and  **FGFR3**  was most pronounced in chondroblasts and hypertrophic chondrocytes, while FGFR2 immunoreactivity was strongest in the articular and prechondroblastic zones.  Ref: 12243968 Arch Oral Biol, 2002
- Standard in situ immunohistochemical techniques were used to stain each cochlea for FGF 1  [ **FGF1** ]  and  **FGFR3** , apoptotic markers p53 and p21, Bcl 2, and the cell cycling marker proliferating cell nuclear antigen PCNA.  Ref: 12512896 J Otolaryngol, 2002
- Formalin fixed, paraffin embedded normal tissues and 60 well characterized GEP endocrine tumors were immunostained using specific antibodies directed against various GEP hormones, aFGF  [ **FGF1** ] , FGFR1, FGFR2,  **FGFR3** , and FGFR4.  Ref: 11759058 Appl Immunohistochem Mol Morphol, 2001
- Moreover, following FGF  [ **FGF1** ]  stimulation, levels of  **FGFR3**  mRNA and protein decline, as does downstream signaling through the MAPK pathway.  Ref: 11861762 J Cell Sci, 2002
- In response to  **FGF1** ,  **FGFR3**  induced a marked cell morphology change to a round shape, while FGFR3DeltaAB did not.  Ref: 11779141 Biochem Biophys Res Commun, 2002
- The objective of this study was to determine whether FGF 1  [ **FGF1** ] , FGF  [ **FGF1** ]  2, and high affinity receptors FGFR2 and  **FGFR3**  are present in the pathogenesis of oral epithelial dysplasias and oral squamous cell carcinoma.  Ref: 12075207 Oral Surg Oral Med Oral Pathol Oral Radiol Endod, 2002
- First, we demonstrated that FGF  [ **FGF1** ]  receptor 1 Fgfr1 and  **Fgfr3** , expressed differentially in the developing brain, possess an equivalent potential to induce the regionally distinct Fgf8 responsive genes, depending on the anterior posterior dimension of the brain.  Ref: 11782403 Development, 2002
- RESULTS A large stromal subpopulation expresses fibroblast growth factor receptor 3  [ **FGFR3** ]  FGF  [ **FGF1** ]  R3, indicating a mesenchymal origin of these cells.  Ref: 12865629 PathobiologyPathobiology, 2002
- Since the presence of FGF  [ **FGF1** ]  receptors FGFRs determines cellular responsiveness, we examined the localisation of FGFR1, FGFR2 and  **FGFR3**  expression by immunohistochemistry throughout the repair of full thickness excisional wounds up to 28 days after wounding.  Ref: 12648559 Arch Oral Biol, 2003
- Immunohistochemical localization of growth factors fibroblast growth factor 1  [ **FGF1** ]  and fibroblast growth factor 2 and receptors fibroblast growth factor receptor 2 and fibroblast growth factor receptor 3  [ **FGFR3** ]  in normal oral epithelium, epithelial dysplasias, and squamous cell carcinoma.  Ref: 12075207 Oral Surg Oral Med Oral Pathol Oral Radiol Endod, 2002
- Point mutations in the human fibroblast growth factor FGF  [ **FGF1** ]  receptor 3 gene  **Fgfr3**  produce a constitutively active receptor, which disrupts chondrocyte differentiation in the growth plate and results in skeletal dysplasias with severe shortening of the limbs.  Ref: 11772395 Biochem J, 2002
- Immunoreactivity of both FGF 1  [ **FGF1** ]  and  **FGFR3**  was observed in spiral ganglion neurons, inner and outer hair cells, pillar cells, and Dieter and Hensen s cells.  Ref: 12512896 J Otolaryngol, 2002
- In complementary in vitro assays, bFGF did not directly stimulate avian OC development from bone marrow mononuclear cell precursors, consistent with their low mRNA expression of the four avian signaling FGF  [ **FGF1** ]  receptors FGFR 1, FGFR 2, FGFR 3  [ **FGFR3** ] , and FGFR like embryonic kinase FREK.  Ref: 12369790 J Bone Miner Res, 2002
- STUDY DESIGN Sections from formalin fixed, paraffin embedded samples of oral normal mucosa n = 14, epithelial dysplasia n = 20, carcinoma in situ n = 10, and squamous cell carcinoma n = 12 were tested for cytoplasmic staining by standard in situ immunohistochemistry with antibodies for FGF 1  [ **FGF1** ] , FGF  [ **FGF1** ]  2, FGFR2, and  **FGFR3** .  Ref: 12075207 Oral Surg Oral Med Oral Pathol Oral Radiol Endod, 2002
- Fibroblast growth factor receptor 3  [ **FGFR3** ]   **FGFR3**  is one of the membrane bound tyrosine kinases that mediate the actions of fibroblast growth factor FGF  [ **FGF1** ]  family members.  Ref: 12687892 Pediatr Pathol Mol Med< MedlineTA>Pediatr Pathol Mol Med, 2002
- The effects of increased  **FGFR3**  activation were determined by exposing cochlear explants to FGF2, a strong ligand for several FGF  [ **FGF1** ]  receptors.  Ref: 12417662 J Neurosci, 2002
- Using semiquantitative reverse transcription polymerase chain reaction, we examined the expression of insulin like growth factor 1 Igf 1, fibroblast growth factor 2 Fgf  [ **FGF1** ]  2, and their receptors Igf 1r, Fgfr1, Fgfr2, and  **Fgfr3**  in the MCC of 28 day old rats at, and 14 days after placing intraoral appliances designed to produce a lateral functional shift of the mandible.  Ref: 12594422 Am J Orthod Dentofacial Orthop, 2003
- This singular response has been linked to signaling via FGF  [ **FGF1** ]  receptor 3  **FGFR3** , partly because mutations causing chronic  **FGFR3**  activation lead to various human disorders of bone growth.  Ref: 11861762 J Cell Sci, 2002
- Upon FGF  [ **FGF1** ]  stimulation,  **FGFR3**  is selectively removed from the focal adhesions, which is followed by their disassembly and disruption of the organized cytoskeleton.  Ref: 11861762 J Cell Sci, 2002
- Activating mutations in FGF  [ **FGF1** ]  receptor 3  **FGFR3**  cause several human dwarfism syndromes by affecting both chondrocyte proliferation and differentiation.  Ref: 12821644 J Cell Biol< MedlineTA>J Cell Biol, 2002
- To understand the biological functions and signal transduction of these  **FGFR3**  isoforms, we analyzed the effect of  **FGF1**  in ATDC5 cells, chondroprogenitor cell lines overexpressing these isoforms.  Ref: 11779141 Biochem Biophys Res Commun, 2002
